# Identification of ferroptosis genes in immune infiltration and prognosis in thyroid papillary carcinoma using network analysis

**DOI:** 10.1186/s12864-021-07895-6

**Published:** 2021-07-27

**Authors:** Ruoting Lin, Conor E. Fogarty, Bowei Ma, Hejie Li, Guoying Ni, Xiaosong Liu, Jianwei Yuan, Tianfang Wang

**Affiliations:** 1 Department of Nuclear Medicine, The First Affiliated Hospital/Clinical Medical School, Guangdong Pharmaceutical University, Guangzhou, 510080 Guangdong China; 2 Genecology Research Centre, University of the Sunshine Coast, Maroochydore DC, QLD 4558 Australia; 3 Department of TCM Resident Training, The First Affiliated Hospital of Guangzhou University of Traditional Chinese Medicine, Guangzhou, 510405 Guangdong China; 4 Cancer Research Institute, First People’s Hospital of Foshan, Foshan, 528000 Guangdong China

**Keywords:** Thyroid papillary carcinoma, Immune-related genes, Tumour microenvironment, Ferroptosis genes, Prognostic model

## Abstract

**Background:**

Papillary thyroid carcinoma (PTC) is the most common thyroid cancer. While many patients survive, a portion of PTC cases display high aggressiveness and even develop into refractory differentiated thyroid carcinoma. This may be alleviated by developing a novel model to predict the risk of recurrence. Ferroptosis is an iron-dependent form of regulated cell death (RCD) driven by lethal accumulation of lipid peroxides, is regulated by a set of genes and shows a variety of metabolic changes. To elucidate whether ferroptosis occurs in PTC, we analyse the gene expression profiles of the disease and established a new model for the correlation.

**Methods:**

The thyroid carcinoma (THCA) datasets were downloaded from The Cancer Genome Atlas (TCGA), UCSC Xena and MisgDB, and included 502 tumour samples and 56 normal samples. A total of 60 ferroptosis related genes were summarised from MisgDB database. Gene set enrichment analysis (GSEA) and Gene set variation analysis (GSVA) were used to analyse pathways potentially involving PTC subtypes. Single sample GSEA (ssGSEA) algorithm was used to analyse the proportion of 28 types of immune cells in the tumour immune infiltration microenvironment in THCA and the hclust algorithm was used to conduct immune typing according to the proportion of immune cells. Spearman correlation analysis was performed on the ferroptosis gene expression and the correlation between immune infiltrating cells proportion. We established the WGCNA to identify genes modules that are highly correlated with the microenvironment of immune invasion. DEseq2 algorithm was further used for differential analysis of sequencing data to analyse the functions and pathways potentially involving hub genes. GO and KEGG enrichment analysis was performed using Clusterprofiler to explore the clinical efficacy of hub genes. Univariate Cox analysis was performed for hub genes combined with clinical prognostic data, and the results was included for lasso regression and constructed the risk regression model. ROC curve and survival curve were used for evaluating the model. Univariate Cox analysis and multivariate Cox analysis were performed in combination with the clinical data of THCA and the risk score value, the clinical efficacy of the model was further evaluated.

**Results:**

We identify two subtypes in PTC based on the expression of ferroptosis related genes, with the proportion of cluster 1 significantly higher than cluster 2 in ferroptosis signature genes that are positively associated. The mutations of *Braf* and *Nras* are detected as the major mutations of cluster 1 and 2, respectively. Subsequent analyses of TME immune cells infiltration indicated cluster 1 is remarkably richer than cluster 2. The risk score of THCA is in good performance evaluated by ROC curve and survival curve, in conjunction with univariate Cox analysis and multivariate Cox analysis results based on the clinical data shows that the risk score of the proposed model could be used as an independent prognostic indicator to predict the prognosis of patients with papillary thyroid cancer.

**Conclusions:**

Our study finds seven crucial genes, including *Ac008063.2*, *Apoe*, *Bcl3*, *Acap3*, *Alox5ap*, *Atxn2l* and *B2m,* and regulation of apoptosis by parathyroid hormone-related proteins significantly associated with ferroptosis and immune cells in PTC, and we construct the risk score model which can be used as an independent prognostic index to predict the prognosis of patients with PTC.

**Supplementary Information:**

The online version contains supplementary material available at 10.1186/s12864-021-07895-6.

## Introduction

Thyroid cancer (TC) is the most common endocrine malignant tumour worldwide and its incidence has been increasing [1]. In 1990, there were an estimated 95,030 incident cases of TC and 22,070 deaths; this increased to 255,490 incident cases and 41,240 deaths in 2017 [2]. In 2020, TC has been found in 9th place for incidence among all cancers, with more than 586,000 cases diagnosed worldwide. In addition, there is a 3-fold higher incidence rate of TC in women, which is about 10.1 per 100,000 cases, representing one in every 20 cancers diagnosed among women [3]. TC shows the highest incidence rates in Northern America, Australia/New Zealand, Eastern Asia, and Southern Europe. The incidence rate of TC is also increasing in China, and it has become one of the ten major cancers threatening the health of Chinese residents [4]. The rapid increase of thyroid cancer has been largely attributed to the use of progressively sensitive diagnostic imaging modalities [5, 6]. A study also found that obesity positively correlated with 16% of TC cases and 63% of large-size tumors diagnosed from 2013 to 2015 in the USA [7]. Differentiated thyroid cancer, which originates from thyroid follicular epithelial cells, accounts for more than 95% of TC, among which papillary thyroid carcinoma (PTC) constitutes more than 85% of cases [8, 9].

Traditional treatment methods of PTC include radical surgery, endocrine therapy and ^131^I therapy, and most treatments display relatively good efficacy, e.g., patients who are disease-free after one course of ^131^I ablation show a range of in 1–3% recurrence rate [10]. However, a small proportion of PTC displays high aggressiveness and even develops into RAI-refractory differentiated thyroid cancer (RAIR-DTC). This causes roughly 15% of the patients to experience recurrence within 10 years after the initial treatment [11–13]. Accurate assessment of the prognosis of PTC is essential to ensure that high-risk and advanced patients receive appropriate treatment without over-treating low-risk patients. The American Thyroid Association (ATA) currently recommends the use of TNM staging to predict mortality and proposes a system to assess the risk of recurrence, which includes the size of the main tumour and whether it has grown into nearby areas (T), the extent of spread to nearby lymph nodes (N), and whether the cancer has metastasised to other organs of the body (M) [9, 14]. It has been addressed that tumour microenvironment (TME) possibly plays an important role in response to chemotherapy and antiangiogenic therapy [15, 16]. In the process of tumorigenesis, tumour cells interact with the surrounding microenvironment to promote immune tolerance, which then develops into immune escape that eventually results in tumour development and angiogenesis, invasion, metastasis, and chronic inflammation [10].

TME has been found to be a complex and continuously evolving matrix, containing such cells as stromal cells, fibroblasts, endothelial, innate and adaptive immune cells [17]. The transcriptome of PTC has been well-characterised and the major molecular events associated with most PTC cases, including the aberrant expression of *Brafv600e*, *Ras*, point mutations of *Tert* and RET/PTC rearrangements were elucidated [18–20]. However, reports on the interaction between PTC and stromal tissue, lymphocytic infiltrate, or normal thyrocytes and expression profiles of associated marker genes are limited. A study of 23 PTC patients using oligonucleotide microarrays identified 19 genes differentially expressed with reference to 10 patients with other thyroid disease, such as *Lrp4*, *Eva1*, *Tmprss4*, *Qpct*, and *Slc34a2* [21]. A recent study comparing the gene expression profiles of PTC and normal thyroid in both micro-dissected cells and whole tissue slides revealed crosstalk between cancer cells and TME possibly involving the functions of *Ptcsc*, *Ctgf*, *Tff3*, *Fn1*, *Mpped2* [22].

As a type of programmed cell death, ferroptosis is dependent on iron and induced by the accumulation of oxidatively damaged phospholipids, associated with the malfunction of glutathione-dependent antioxidant defences that are mediated by glutathione peroxidase 4 (*Gpx4*) via different pathways [23–25]. and, resulting in a large amount of ROS, which promotes ferroptosis The declines in metabolism of lipid peroxides catalysed by *Gpx4* and glutathione (GSH) level intracellularly, lead to Fe^2+^ oxidising lipids in a Fenton-like manner, which enhances ferroptosis due to the elevation of lipid reactive oxygen species (ROS) in cells [24, 26]. In recent years, the induction of ferroptosis has been investigated as an alternative and/or joint therapeutic approach to trigger cancer cell death, with respect to other types of cell death, especially for the treatment of malignancies resistance issues in some cancers; the marker genes and modulator molecules of ferroptosis have been identified [13, 27, 28]. However, the role of ferroptosis in PTC currently remains elusive.

In this study, we integrated the THCA dataset from TCGA and the clinical databases of THCA from UCSC Xena to identify reliable differentially expressed genes (DEGs) relevant to ferroptosis in PTC. Then, univariate Cox survival analysis and lasso Cox regression analysis were performed to identify DEGs of PTC, and we proposed a prognostic prediction model using DEGs and clinical data from the TCGA-THCA and UCSC Xena datasets. We performed Multivariate Cox survival analysis on hub genes combined with clinical prognosis data and showed that the model can be used to predict PTC prognosis.

## Methods

### Data processing

The papillary thyroid carcinoma (THCA) datasets were obtained from two platforms. THCA database level 3 count was searched in The Cancer Genome Atlas (TCGA) database (https://portal.gdc.cancer.gov/repository). The datasets in TCGA were transformed into Transcripts Per Million (TPM) values and the mutation data of The Single Nucleotide Polymorphism Database (SNP) were downloaded for THCA. The clinical data of THCA were obtained using the UCSC Xena browser (https://xenabrowser.net/). Only patients with primary tumours who did not receive neoadjuvant therapy were included in this retrospective analysis [13]. Their clinicopathological, genetic, epigenetic, and survival data were downloaded for a secondary analysis. In this study, the datasets of 502 tumour samples and 56 normal samples were used.

### Identification of THCA subtypes based on related genes of ferroptosis

A set of 60 related genes of ferroptosis genes were summarised from the MisgDB database (https://www.gsea-msigdb.org/gsea/msigdb). The ConsensusClusterPlus package was used for consensus clustering and molecular subtype screening. In brief, k-means clustering was used, with 50 iterations (each using 80% of the samples) [29]. The best cluster number was determined by the clustering score for the cumulative distribution function (CDF) curve, and the relative changes in the area under the CDF curve were evaluated. Principal component analysis (PCA) and *t*-distributed Stochastic Neighbor Embedding (t-SNE), commonly used for dimensionality reduction, were used to verify the reliability of the consensus clusters.

### Heatmap

The ssGSEA score x_i_ for each THCA sample i was converted to x_i_′ using the equation:

$$ {x}_i^{\prime }=\left({x}_i-{x}_{min}\right)/\left({x}_{max}-{x}_{min}\right) $$

while *x*_*max*_ and *x*_*min*_ represent the maximum and minimum single-sample gene-set enrichment analysis (ssGSEA) scores for all samples in the THCA dataset, respectively. The relationship among subtypes, clinical parameters and ferroptosis genes gene expression were shown by thermography with R-package pheatmap.

### Comparison of immune cell subgroups among molecular subtypes of THCA

We continued to use maftools to explore the difference between two subtypes and drew the waterfall of them. In order to investigate the biological difference and understand the different pathways involved by the subtypes, Gene Set Enrichment Analysis (GSEA) was used with the clusterProfiler package. Gene set variation analysis (GSVA) was used to further analyze the difference of pathways between the subtypes using GSVA package [30, 31].

### Immune infiltration microenvironment in THCA

The difference between the subtypes may be due to the complexity of tumour microenvironment, so we used the ssGSEA algorithm to analyse the proportion of 28 kinds of immune cells in THCA, and used hclust algorithm to classify the immune cells according to the proportion of immune cells. Finally, a box-plot was constructed to contrast immune cell content between the subtypes. If the normal distribution and homogeneity of variance were met, T-test was used; if not, Wilcox test was used. The above operations were completed by using complexheatmap package, ssGSEA package, corrplot package and ggplot2 package.

### Gene co-expression network analysis

To investigate characteristic immune gene distribution in each molecular subtype of THCA and identify genes or gene modules highly related to immune cell infiltration, the WGCNA R package was used to evaluate 502 immune-related genes comprising the expression matrix. WGCNA network was constructed and the significant modules were identified. Genes of the significant modules were selected as subtype related hub genes for follow-up analysis.

### Data validation of the differentially expressed genes (DEGs)

To further analyse DEGs, DEseq2 algorithm was used for differential analysis of sequencing data (using adjust *P*-value < 0.05 and logFC > 2 as thresholds). Volcano plots and heatmap were applied to visualize the distribution of the overlapping DEGs between the training and test sets [32]. The above operation was done using the DEseq2 package.

### Functional enrichment, pathway analysis of hub genes

In order to identify the possible functions and pathway of hub genes, Gene Ontology (GO) analysis and Kyoto Encyclopedia of Genes and Genomes (KEGG) pathway [33, 34] enrichment analyses were performed using the clusterProfiler package [35]. The visualisation module of clusterProfiler was used for displaying analysis results. *P* < 0.05 was selected as the cut-off criterion.

### Cox regression and survival analysis

To explore the clinical efficacy of hub genes, univariate Cox analysis was performed on hub genes combined with clinical prognosis data, and the results of univariate Cox regression (*P* < 0.05) were included in lasso regression. The lasso regression results were incorporated into multivariate Cox regression. The risk regression model was built according to the multivariate analysis results, and the formula of the model was as follows:

$$ risk\ score=\sum \limits_{i=1}^n{\beta}_i\times {x}_i $$

ROC curve and survival curve were used to evaluate the results of the model. Univariate Cox analysis and multivariate Cox analysis were performed by combining THCA clinical data and risk score values for further evaluation.

### Statistical analysis

All the above analyses were completed by R software. Adjust *P*-value < 0.05, *P*-value < 0.05 and FC > 2 were used as statistical thresholds. The statistical methods and algorithms used are described in the corresponding steps.

## Results

### Identification of THCA subtypes based on immune gene sets

**Fig. 1:**
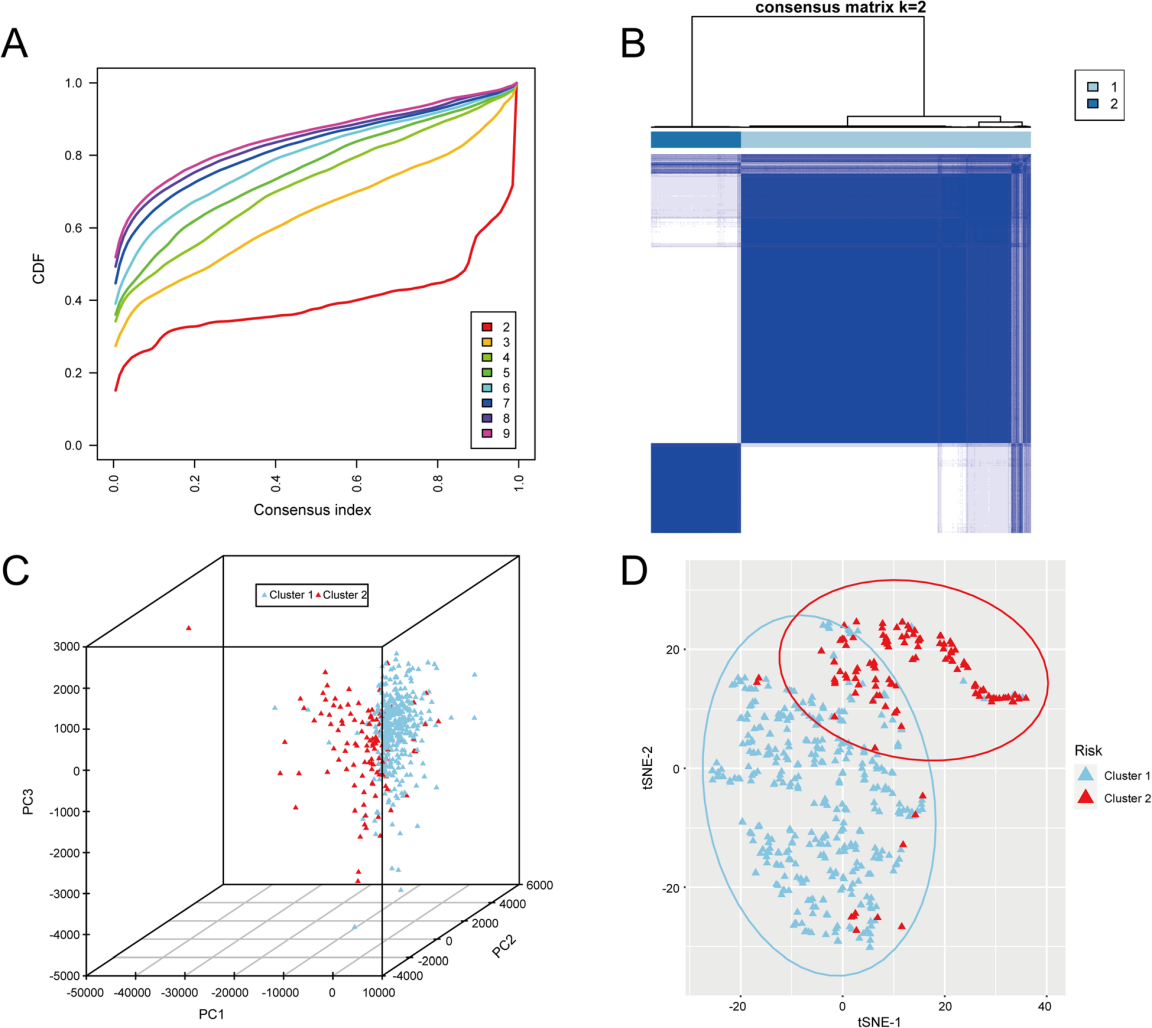
Consensus clustering of THCA TCGA cohorts. **A** CDF curve of the consistency score for different subtype numbers (k = 2–9). **B** The consensus score matrix for THCA samples when k = 2. A higher consensus score between two samples indicates that they are more likely to be assigned to the same cluster in different iterations. **C** Principal component analysis (PCA) plot of the TCGA cohort. **D** 2D t-SNE analysis of the TCGA cohort

The 502 tumour samples of THCA were divided into k subtypes (k = 2–9) using the R package ConsensusClusterPlus. Consensus distributions for each k were displayed, which dissect the optimal k value at which the sample distribution was stable (Fig. 1A). Based on the consensus score of the CDF curve, the optimal cluster number was determined as k = 2, and the relative changes in the area under the CDF curve were evaluated. These were finally divided into two distinct and nonoverlapping subtypes, i.e., cluster 1 and cluster 2. The consensus matrix heat map of these two clusters was shown in Fig. 1B. A principal component analysis (PCA) plot (Fig. 1C) and a two dimensional t-SNE analysis (Fig. 1D) further verified the LUAD cohort and the reliability of the consensus clusters.

### Association among subtypes, clinical parameters and ferroptosis signature genes

**Fig. 2:**
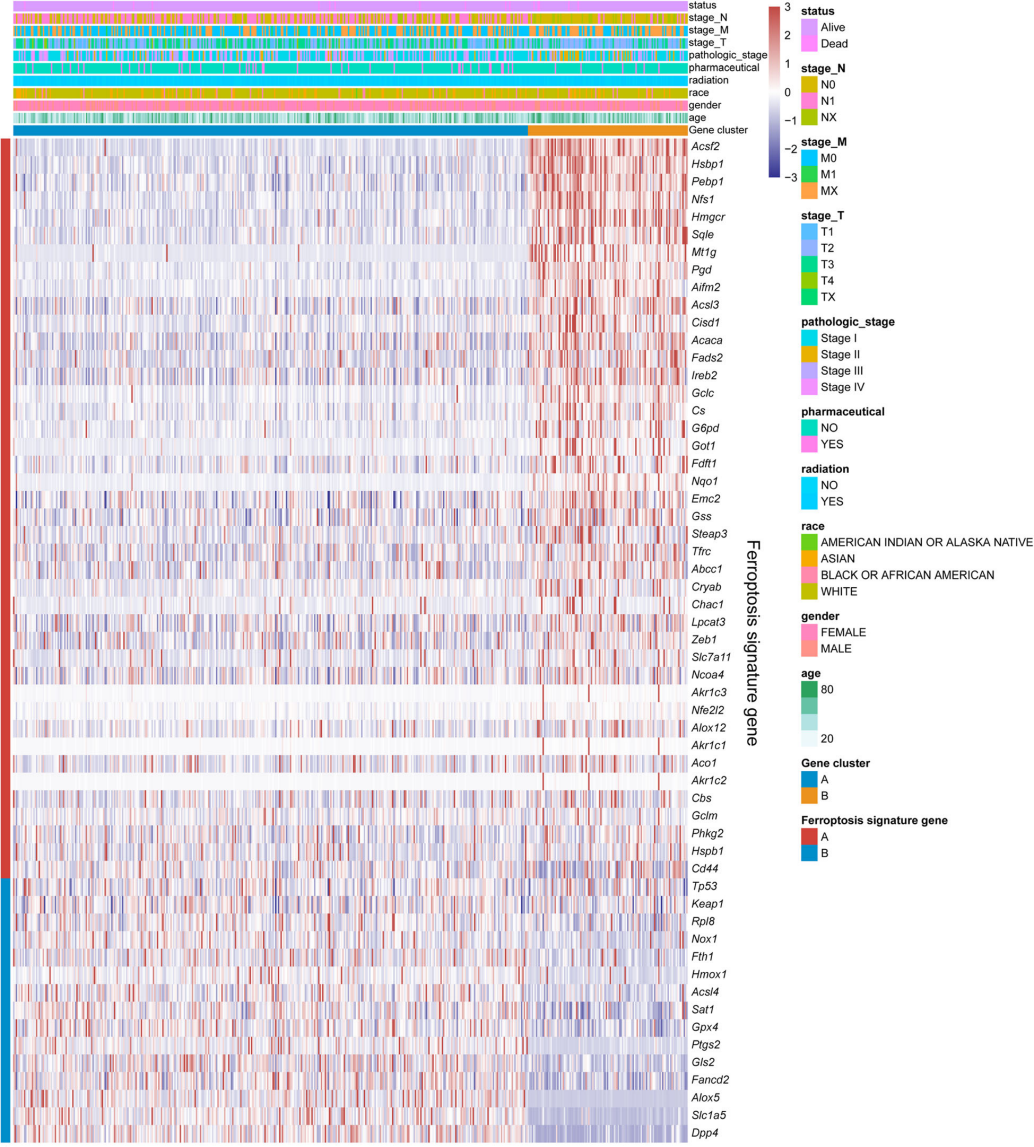
Unsupervised clustering of ferroptosis genes expression in the THCA cohort. The gene cluster, tumour stage, survival status, pathologic stage, pharmaceutical, radiation, race, gender, age, and ferroptosis signature genes were used as patient annotations. Red represented high expression of regulators and blue represented low expression

To explore the association among subtypes, clinical parameters and ferroptosis signature genes, we analysed gene cluster, tumour stage, survival status, pathologic stage, pharmaceutical, radiation, race, gender, age, and ferroptosis signature genes in the TCGA cohort. The relationship among subtypes, clinical parameters and ferroptosis signature genes is derived by thermography (Fig. 2). On the left side of the heatmap, genes that were positively associated with ferroptosis were coded red (such as *Csf2*, *Hsbp1*, *Pebp1*, *Nfs1*, *Hmgcr*, *Sqle*, *Mt1g*, and *Pgd*), whereas blue colour was used to indicate genes that are negatively associated with ferroptosis (incl. *Tp53*, *Keap1*, *Rpl8*, *Nox1*, *Fth1*, *Hmox1*, *Acsl4*, *Sat1*, *Gpx4*, *Ptgs2*, *Gls2*, *Fancd2*, *Alox5*, *Slc1a5* and *Dpp4*). Gene cluster differed significantly between the two subtypes. In particular, the proportion of cluster 2 was significantly higher than cluster 1 in ferroptosis signature genes that are positively associated, yet the expression of these genes generally appeared higher in cluster 2, such as *Acsf2*, *Mt1g*, *Gclc* and *Aifm2*. Moreover, cluster 1 had remarkably larger proportion of genes that are negatively associated with ferroptosis, for instance, *Tp53*, *Dpp4*, *Slc1a5*, *Alox5* and *Ptgs2*.

In terms of the spread magnitude to nearby lymph nodes (stage_N), N1 was largely relevant to cluster 1, while N0 and NX were more present in cluster 2. The metastasis to distant sites (stage_M) showed an increased proportion of MX in cluster 2, while the extent of the tumour (stage_T) distributed across both cluster 1 and 2, with latter exhibited higher proportion of T2 (Fig. 2). There were more pathological stage III and IV cases associated with cluster 1, whereas cluster 2 subtype showed more genes associated with stage II, which represents non-invasive tumour with no spread to lymph nodes and no metastasis.

### Somatic mutation landscape of the two subtypes were identified by GSEA

**Fig. 3:**
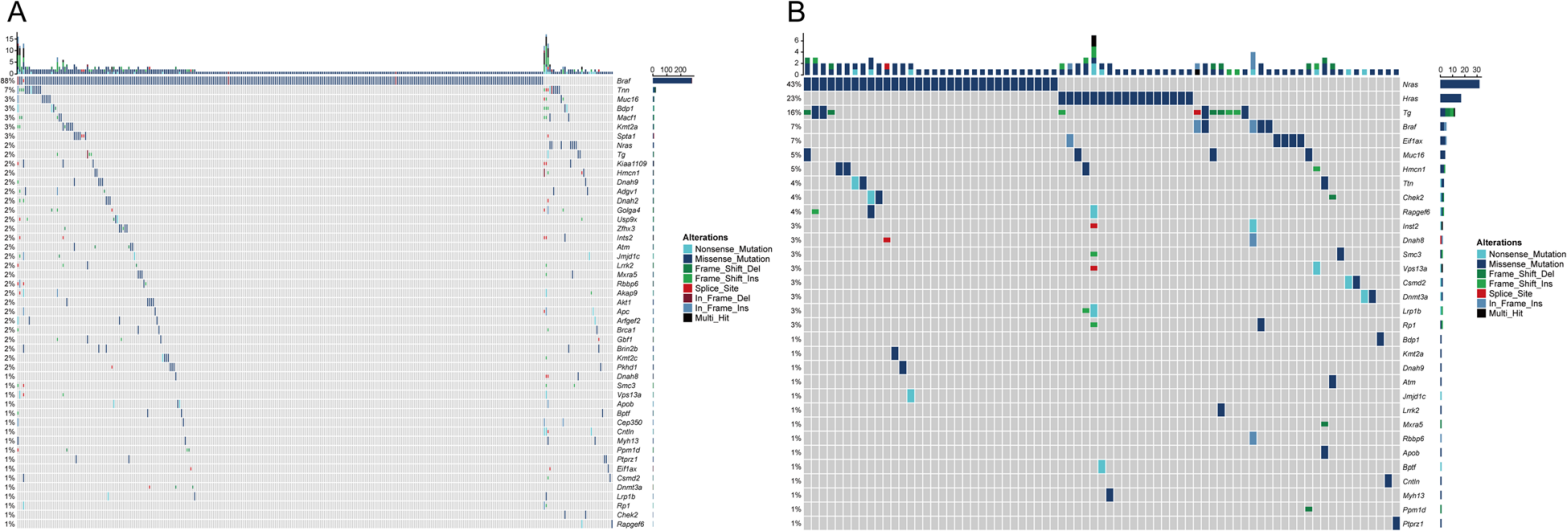
Somatic mutation landscapes of subtypes cluster 1 (**A**) and 2 (**B**) identified by GSEA in TCGA cohort. Landscape of mutation profiles in THCA samples. A total of 34 genes with more than 10% of mutation frequency were chosen in the waterfall plot. Various colours of the waterfall plot with annotations at the bottom represent different mutation types. The barplot above the legend shows the mutation number

We continued to explore the differences between the two subtypes using the maftools package to explore the mutation profile and drawing the waterfall map of them (Fig. 3). It was found that the somatic mutation types of the two subtypes were missense mutations. The top 2 somatic mutation genes of subtype cluster 1 were *Braf* (88%) and *Tnn* (7%) (Fig. 3A), with the other 47 genes with low mutation frequency less than 5%. Cluster 2 showed somatic mutations of *Nras*, *Hras* and *Tg* with more than 10% of mutation frequency (Fig. 3B). This indicates biological differences between the two subtypes.

### Gene set enrichment analysis

**Fig. 4:**
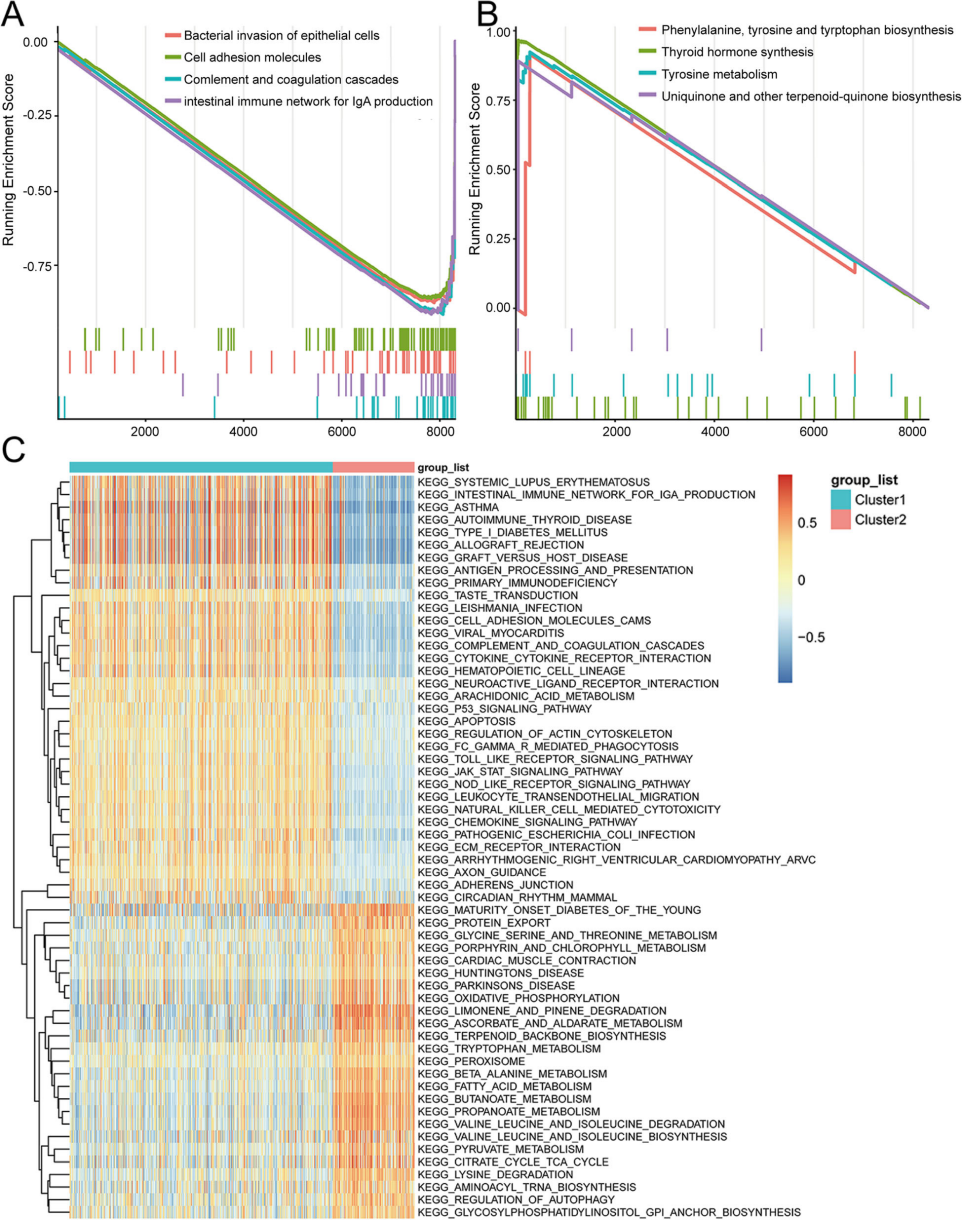
**A** The enriched KEGG pathways associated with DEGs of cluster 1 predicted by GSEA analysis. **B** The enriched KEGG pathways associated with DEGs of cluster 2 predicted by GSEA analysis. **C** Thermogram shows the activation state of KEGG pathways in different clusters after processing by GSVA. The red node represents up-regulation and the blue node represents down-regulation, *P*-value < 0.05

GSEA and GSVA were used to analyse the enriched pathways in each cluster to investigate the molecular differences in the phenotypes of these two subtypes. GSEA analysis suggested that subtype cluster 1 was mainly involved in bacterial invasion of epithelial cells, cell adhesion molecules, completion and coaggregation cascades and intestinal immune network for IgA production (Fig. 4A). In subtype cluster 2 phenylalanine, tyrosine and tryptophan biosynthesis, thyroid hormone synthesis, tyrosine metabolism, ubiquinone and other terpenoid−quinone biosynthesis were enriched (Fig. 4B). GSVA was used to further explore the differences in participating KEGG pathways between the two subtypes, and the results were shown in Fig. 4C (adjust *P*-value< 0.05; see Additional file 1: Table S1 for more details). In addition, there were more immune response relevant pathways present in cluster 1 than cluster 2, such as antigen processing and presentation (*P*-value = 1.27E-22), natural killer cell mediated cytotoxicity (*P*-value = 1.36E-37), Fc gamma R-mediated phagocytosis (*P*-value = 1.12E-23), cytokine-cytokine receptor interaction (*P*-value = 7.18E-45), and toll-like receptor signalling pathway (*P*-value = 1.17E-24). Cluster 2 was enriched with more metabolism pathways, for instance, metabolisms of glycine, serine and threonine (*P*-value = 1.04E-30), porphyrin and chlorophyll (*P*-value = 2.93E-33), and fatty acid (*P*-value = 4.05E-41).

### Immune characteristics of the two subtypes

**Fig. 5:**
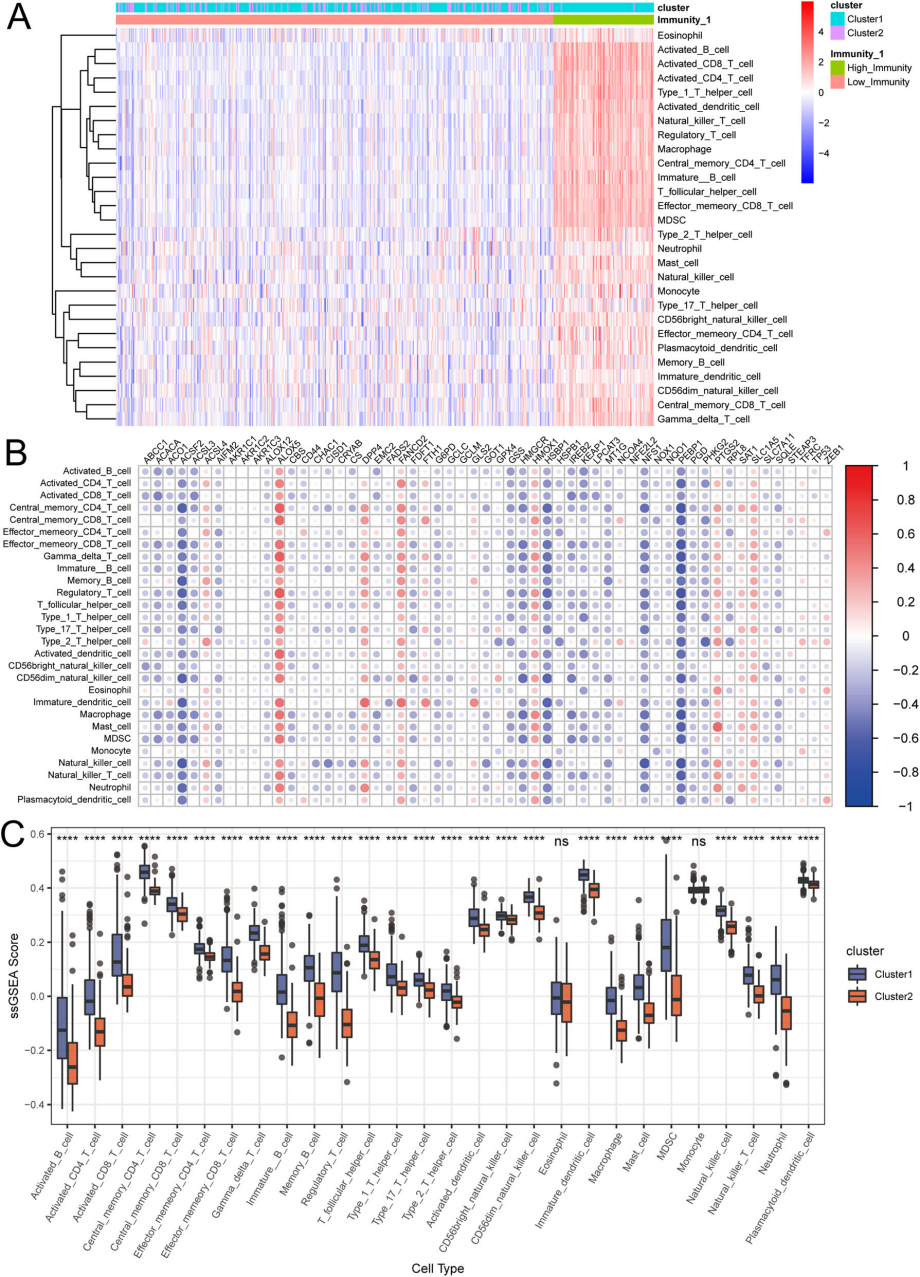
Identification of the two immune subtypes in the THCA cohort. **A** Heatmap of the two immune subtypes based on ssGSEA algorithm for 28 immune gene sets. **B** The correlation heatmap demonstrates the relationship between ferroptosis genes expressions and immune cells infiltration. Dots size shows the extent of their relationships and dots colour indicates if they are positive-related (red dots) or negative-related (blue dots). The number on the scale bar indicates the coefficients of correlation between genes expression and cells infiltration. Blank squares mean insignificance (*P*-value > 0.05). **C** The abundance of each TME infiltrating cell in THCA modification patterns. The upper and lower ends of the boxes represented interquartile range of values. The lines in the boxes represented median value, and black dots showed outliers. The asterisks represented the statistical *P*-value (*P < 0.05; **P < 0.01; ***P < 0.001; ****P < 0.0001)

The difference between two immune subtypes might be formed because of the complexity of the TME. To explore the biological behaviours between the two clusters, we performed ssGSEA algorithm to analyse the proportion of 28 kinds of immune cells in immune infiltration microenvironment in THCA. The results showed that the degree of immune infiltration in cluster 1 was largely higher than in cluster 2, suggested by the greater activation of immune response relevant cells, among which activated B cells, CD8 and CD4 T cells appeared to be the top three cell types (Fig. 5A). Spearman correlation analysis was used to calculate the correlation between ferroptosis gene expression and the proportion of immune infiltrating cells (Fig. 5B). This showed positive correlation of *Alox5*, *Fancd2*, *Dpp4*, *Hmox1*, *Ptgs2*, *Slc1a5*, *Sat1* and *Acsl4* with most cell types with relatively high magnitudes. In contrast, the negative correlation of these cells with ferroptosis was indicated by the relative low expression of *Acsf2*, *Pebp1*, *Nfs1*, *Hsbp1*, *Hmgcr*, *Acsl3* and *Gss*. The difference of immune cell contents between the two subtypes were compared (Fig. 5C and Additional file 2: Table S2). Notably, cluster1 was remarkably enriched (*P*-value < 0.0001) with nearly all innate immune cell types, except eosinophil and monocyte, compared to cluster 2.

### Identification of DEGs distinct phenotypes derived from gene co-expression network

**Fig. 6:**
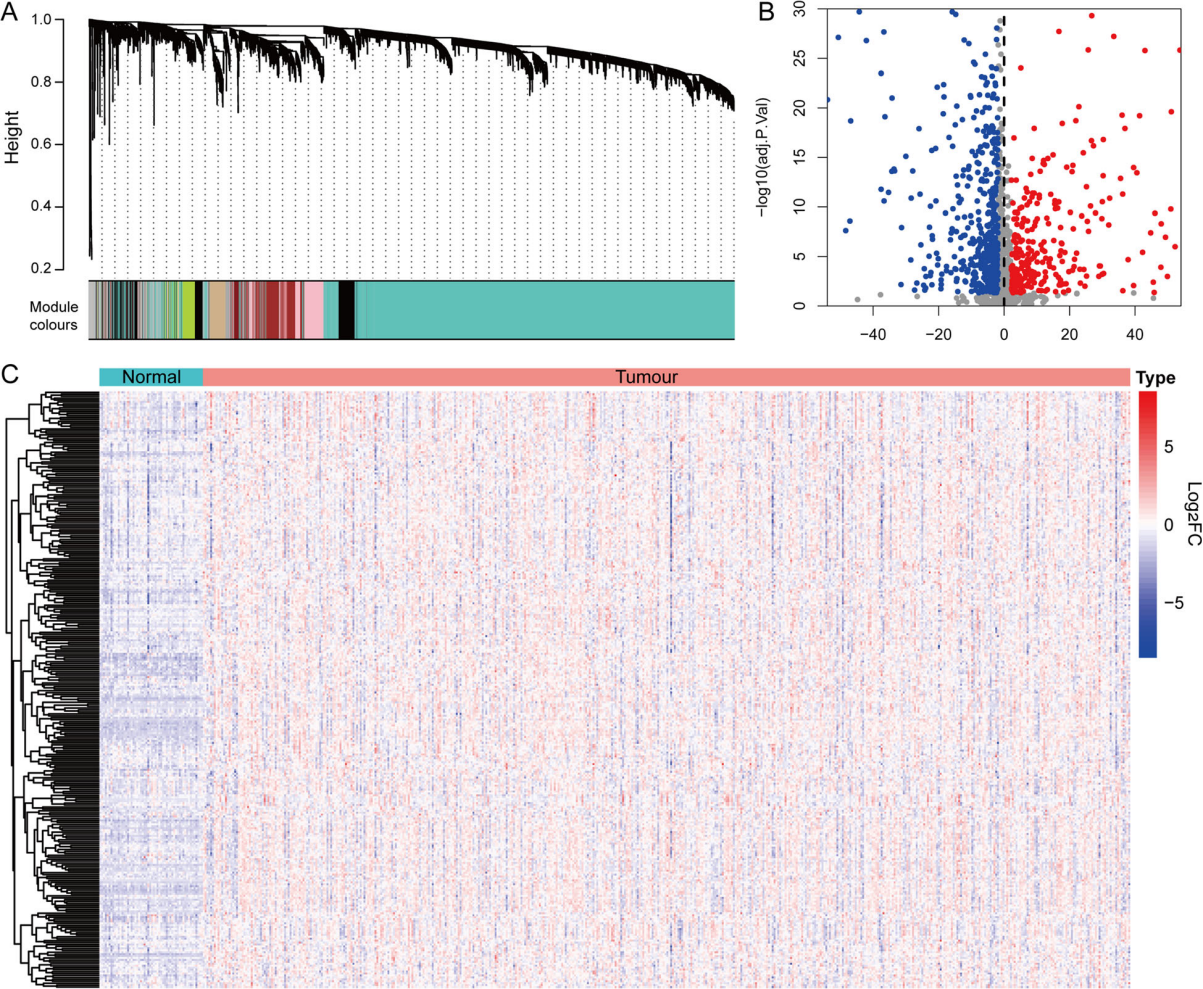
**A** Gene dendrogram and module colours of 502 PTC patients. **B** The volcano plot and **C** heatmap plot of 814 hub genes differentially expressed in tumour and normal tissues (including 308 up-regulated and 506 down-regulated genes)

The distribution of genes expression profiles in the subtypes was investigated to identify genes or gene modules highly related to immune infiltration microenvironment. The WGCNA R-package was used to evaluate the expression matrix data from 502 tumour samples. Analysis of network topology for various soft-thresholding powers indicated that when the power value was equal to 5 (β = 5), the predicted gene co-expression network exhibited scale-free topology by the fit index greater than 0.8, with inherent modular features (Additional file 3: Fig. S1A). The adjacency function was used to generate the adjacency matrix based on the β and gene expression matrix. The hierarchical clustering was built based on the TOM dissimilarity measure (Fig. 6A). A total of 25 seven co-expression modules were detected. The module preservation statistics was employed to achieve reliable and preserved modules (Fig. S1B). The co-expression network was examined by the NULMS Stanford dataset, with genes assigned to modules based on the modules in the reference dataset. There are 16 modules strongly preserved (Z-summary more than 10), such as blue, green, turquoise yellow and red modules; while seven modules are moderately preserved (5 < Z-summary < 10).

The association between module eigengenes, subtypes and immunity were then computed through the Pearson’s correlation coefficient, which evaluates the *P*-value was calculated for any given correlation. The blue module was most significantly correlated with subtypes, and the yellow module had the highest correlation with immune typing (Additional file 3: Fig. S1C). Therefore, the genes of these two modules were selected as subtype related hub genes for subsequent analysis, which included a total of 1747 hub genes. The DEseq2 algorithm was used to further analyse the hub genes in the sequencing data, and significant differentially expression of 814 hub genes were found, among which 308 were up-regulated and 506 were down-regulated in tumour tissues (Fig. 6B). The heat map comparing the expression of these 814 genes in Log2FC values was displayed in Fig. 6C (See Additional file 4: Table S3 for the full gene list).

### Gene ontology and KEGG pathway analysis showed functional enrichment in immune regulations

**Fig. 7:**
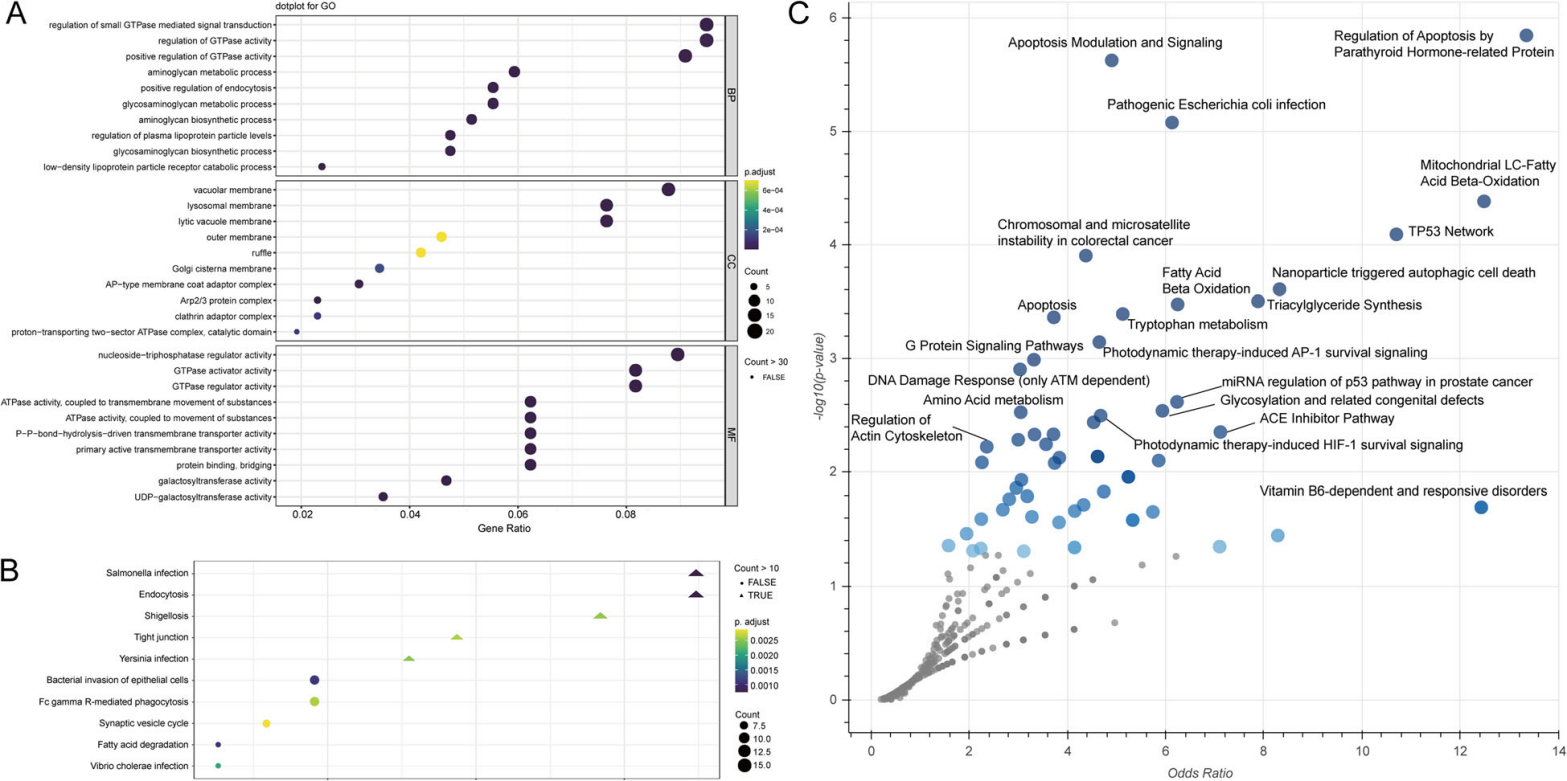
**A** The GO analysis and **B** the KEGG pathway enrichment analysis of 814 hub genes. The node colour changes gradually from yellow to black in ascending order according to the adjusted *P*-values. The size of the node represents the number of counts. **C** The Wikipathway enrichment analysis. Larger blue points represent significant terms (*P*-value < 0.05); smaller grey points represent non-significant terms. The darker the blue colour of a point, the more significant it is

To further analyse the functions and pathways that 814 hub genes are potentially involved in, GO term and KEGG pathway enrichment analyses were performed using the clusterProfiler package. GO analysis showed that 814 hub genes were mainly involved in biological processes such as regulation of small GTPase mediated signal transduction, positive regulation of endocytosis and regulation of plasma lipoprotein particle levels. In terms of cellular components, these genes showed high relevance to membrane (Fig. 7A). KEGG pathway analysis showed that 814 hub genes were mainly enriched in Salmonella infection and endocytosis pathways (Fig. 7B). The enrichment analysis of Wikipathway revealed that regulation of apoptosis by parathyroid hormone-related protein, apoptosis modulation and signalling, and pathogenic *Escherichia coli* infection were the top 3 pathways (Fig. 7C). Besides, several cancer relevant pathways were also enriched in the hub genes (Additional file 4: Table S3).

### Establishment and assessment of the predict model

**Fig. 8:**
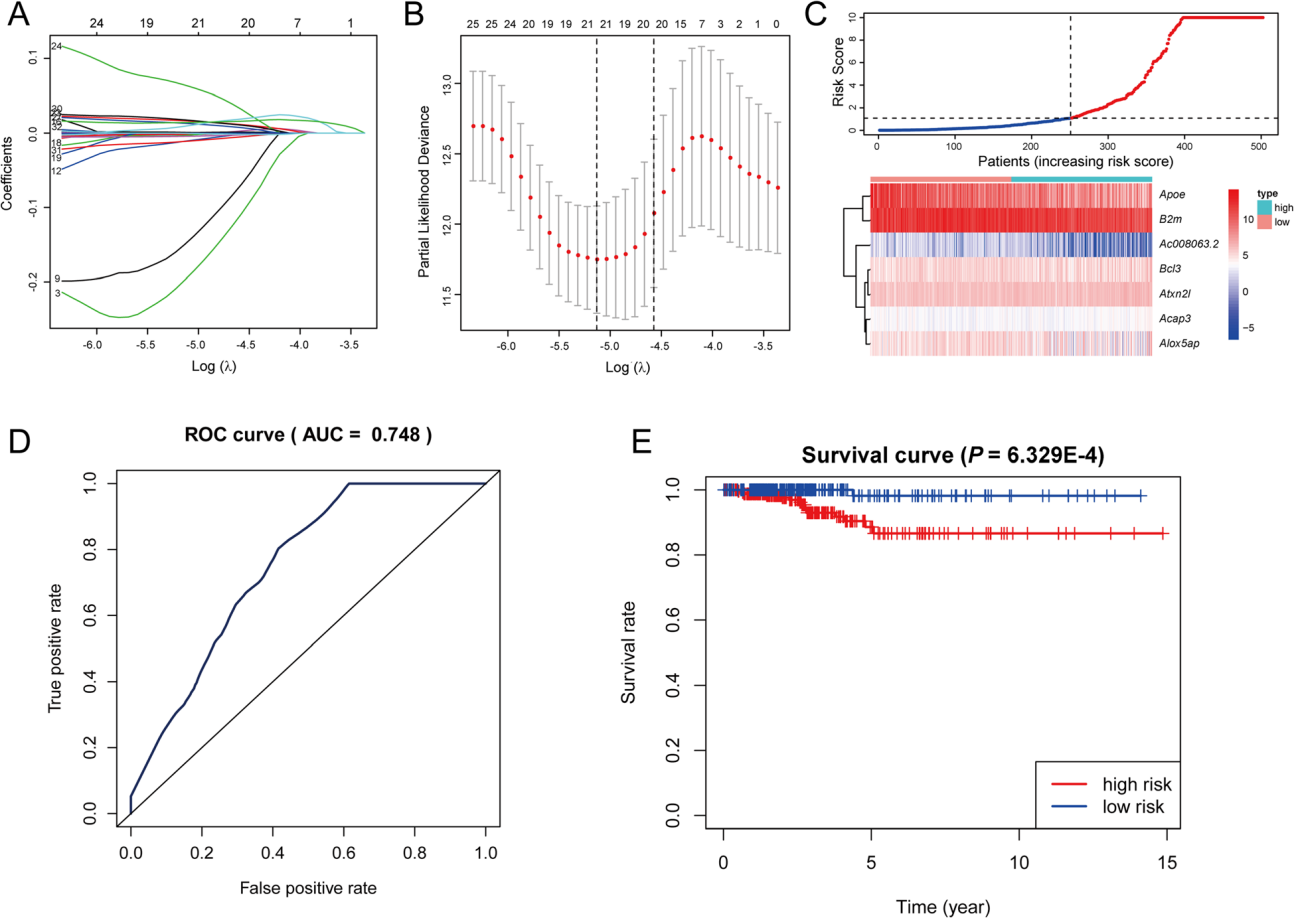
**A** Log (Lambda) value of the 24 genes in LASSO model. **B** Cross-validation for tuning parameter selection in the proportional hazards model, dotted vertical lines were drawn at the optimal values, and seven genes were identified by LASSO regression. **C** The distribution of risk score and gene expression levels among patients. **D** The ROC curve for assessing the reliability of the predict model. **E** The Kaplan-Meier curve of the predicted model

To explore the clinical efficacy of 814 hub genes, Univariate Cox analysis was performed with clinical prognosis data and the results of *P*-value < 0.05 were recorded in Table S3. The results of Univariate Cox regression (*P*-value < 0.05) were included in lasso regression. Dimensionalisation was reduced according to the Lambda curve (Fig. 8A) and the proportional hazards model curve (Fig. 8B), which showed that the deviance was the smallest when the number of genes in the model was 19. The lasso regression results were incorporated into multivariate Cox regression, as seven genes were identified by LASSO regression (Additional file 4: Table S3), including *Ac008063.2*, *Apoe*, *Bcl3*, *Acap3*, *Alox5ap*, *Atxn2l* and *B2m*. The risk regression model diagram was displayed in Fig. 8C according to the multivariate analysis results. In order to evaluate the specificity and sensitivity of the prognostic model, ROC curve and survival curve were used for evaluation. The AUC value of the ROC curve was 0.748 (Fig. 8D) and the survival curve showed a significant difference between the high-risk and low-risk groups (*P*-value = 6.329E-4), indicating a good result of the model (Fig. 8E).

### The risk score predicted prognosis

**Fig. 9:**
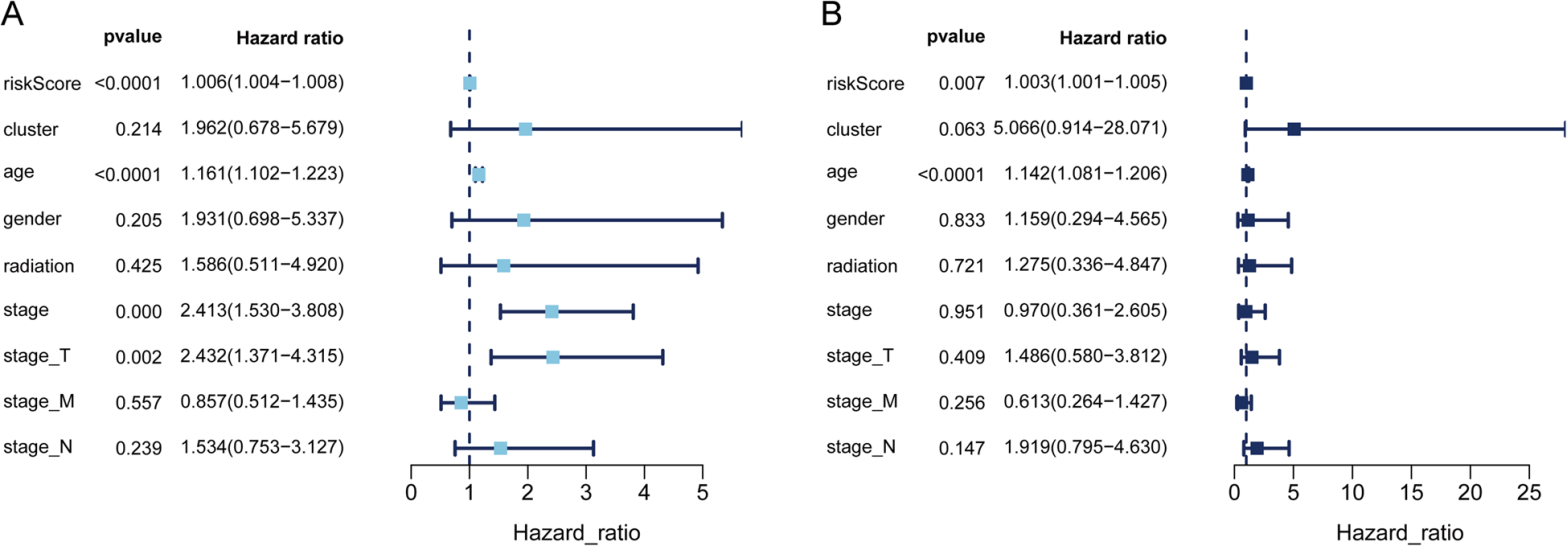
Univariate and Multivariate Cox analysis analysis of risk score, cluster, age, gender, radiation and TNM stage. **A** Prognostic value detection of the gene signature via univariate survival-related analysis in PTC. **B** Prognostic value detection of the gene signature via multivariate survival-related analysis in PTC

To further evaluate the clinical efficacy of the model, univariate Cox analysis (Fig. 9A) and multivariate Cox analysis (Fig. 9B) were performed by combining THCA clinical data and risk score values, which implied that the risk score value of the model could be used as an independent prognostic indicator to predict the prognosis of patients with thyroid papillary carcinoma (see Additional file 5: Table S4 for full statistical results).

## Discussion

The incidence of PTC is a heavy disease burden around the world, and has increased quite significantly over the past two decades [36–38]. Patients with recurrent PTC who undergo surgical treatment have a high risk of complications, including tracheal collapse, laryngeal edema and recurrent laryngeal nerve injury [9, 36, 39]. Furthermore, PTC is a highly heterogeneous disease and tumour progression involves a complex network comprised of multiple signalling pathways [40, 41]. Nomogram is widely used to evaluate clinical prognosis in oncology because it can integrate a variety of prognostic determinants, including molecular biological and clinicopathological parameters [8, 40, 42]. The combination of our prognostic gene signature with conventional clinical parameters may provide a better tool to predict PTC prognosis.

The discovery of distinct lethal subroutines of RCD has facilitated great progress in cancer treatment [43–45]. Ferroptosis was identified as a new form of RCD distinct from apoptosis, necrosis, autophagy, and other forms of cell death. It is an iron-dependent cell death process and caused by lipid peroxidation relying on reactive oxygen species (ROS) generation [24, 46, 47]. There has been growing interest in exploring the mechanisms underpinning ferroptosis in recent years, and several seminal discoveries have elucidated the process [44, 48, 49]. Ferroptosis is thought to only be inhibited by the phospholipid hydroperoxide-reducing enzyme glutathione peroxidase 4 and radical-trapping antioxidants [26, 44]. GSH-GPX4 and FSP1-CoQ10 are the two main ferroptosis resistance pathways [50, 51].

Growing evidence suggests that immune cells in TME play vital roles in tumorigenesis. These innate immune cells include macrophages, neutrophils, dendritic cells, innate lymphoid cells, myeloid-derived suppressor cells, and natural killer cells which potentially possess tumour-antagonising or tumour-promoting functions [29, 52, 53]. Cancer cells exhibited iron ion aggregation in the TME for active proliferation, thus from the perspective of iron homeostasis, regulating ferroptosis could effectively kill tumour cells. In addition, ferroptosis also plays an important immunological role, participating in the process of tumour surveillance by immune cells and tumour immunity [52, 54, 55]. The metabolism of immune cells affects their differentiation and function [56]. Given the complex interaction of environmental factors in TME as a profound impact on the metabolic activities of immunity, matrix and tumour cell types and ferroptosis [48, 57]. Thus, exploring immunophenotype in TME could help reveal the significance of ferroptosis in cancer treatment. The cross study of ferroptosis and immune cells might lead to a discussion of clinical application.

In this study, we seek to investigate whether there was ferroptosis regulation via exploring the crucial genes and pathways of ferroptosis in PTC. A total of 502 PTC samples and 56 normal samples integrated from TCGA and UCSC Xena datasets were analysed, and 60 ferroptosis related genes were derived from MisgDB datasets. Two distinct subtypes (i.e. cluster 1, cluster 2) were identified in our study accordingly based on these related genes. The proportion of cluster 1 was significantly higher than cluster 2 in the signature genes that either positively or negatively associated with ferroptosis. The main mutation types of these two subtypes were identified as missense mutation. The missense mutations of *Braf* have been detected in circulating DNA in the serum of some patients with PTC [58]. A close association of *Braf* mutations with extrathyroidal extension, lymph node metastasis, and stage III/IV of PTC has been strongly suggested [59]. The significance of missense mutations of *Nras* and *Hras* in PTC has also been identified [60–62].

The distinct enriched KEGG pathways revealed GSEA analysis of cluster 1 and cluster 2 implied biological differences between the two genotypes. The GSVA analysis showed that there were more immune response relevant pathways enriched in cluster 1 (adjust *P*-value < 0.05, Additional file 1: Table S1). The difference between the two subtypes possibly resulted from the complexity of TME, since the degree of immune infiltration in cluster 1 was remarkably higher than that in cluster 2, which was reflected by the contents of many immune cell types. Subsequent analysis of TME cell infiltration indicated THCA cluster1 was remarkably rich in innate immune cell infiltration including T cells, natural killer cells, macrophage, eosinophil, mast cell, MDSC, plasmacytoid dendritic cell.

We subsequently analysed the related hub genes of the two modules with the highest correlation between molecular typing and immune typing in WGCNA network, and there were 814 DEGs including 308 up-regulated genes and 506 down-regulated genes in PTC tissues. Then, lasso regression was evaluated and incorporated into multivariate Cox regression, the crucial genes (i.e., *Ac008063.2*, *Apoe*, *Bcl3*, *Acap3*, *Alox5ap*, *Atxn2l* and *B2m*) were identified and the risk regression model was constructed according to the results of multivariate analysis. ROC curve and survival curve indicated a good result of the model. Based on these findings, the risk score of the model could be used as an independent prognostic indicator to predict the prognosis of patients with PTC.

All of these genes, with the exception of *Ac008063.2*, have been implicated in cancer cell proliferation. With the exception of *Apeo*, which is highly expressed in liver cancer, all of the genes have low cancer specificity, according to Human Protein Atlas (*Ac008063.2* could not be identified). *B2m* has been identified as a potential biomarker for thyroid cancer, kidney disfunction and renal disease [63]. Higher rates of *B2m* mutation are correlated with lower patient survival rates, which is speculated to be due to increased immune evasion [64, 65]. *Atxn2l* is a stress response molecule which has been identified as a potential cancer prognosis gene for cancer, such as adrenocortical carcinoma [66, 67]. *Alox5ap* has been identified as specifically upregulated in leukemia stem cells and unchanged in hematopoietic stem cells, indicating specificity in cancer cell self-renewal and differentiation [68, 69]. There is relatively little oncological literature on *Acap3*, however it has been identified as highly down-regulated in late-stage liver cancer patients [70]. *Bcl3* up-regulation has been associated with poorer prognostic outcomes [71, 72]. It is classified as an oncogene and has been implicated in breast cancer cell migration [73, 74]. *Apoe* is associated with tumorigenesis in many cancers, including lung, gastric and thyroid cancer, and a higher risk of metastasis [75–77].

To our knowledge, a prognostic model based on ferroptosis genes and immune cells in tumour immune infiltration microenvironment has not been reported up to now. Compared with whole genome sequencing, our prediction model based on a limited number of gene expression levels might be more economical and practical. Further, the risk score of the model could predict the prognosis of patients with PTC. However, the external validation of the seven-gene signature and prognostic nomogram is needed in more independent cohorts. In addition, to clarify the molecular mechanism underlying the seven genes in relation to PTC, the spatial expression pattern and quantity of these genes at protein level warrants further investigation.

## Conclusions

We demonstrated that ferroptosis was associated with immune cell infiltration in TME of patients with PTC, based on which a seven-gene signature and a prognostic model to predict the prognosis was established. The seven genes appeared closely related to the progression and prognosis of PTC and thus could be potential therapeutic targets. The predicted model proved to be reliable in predicting the prognosis of patients with PTC and might thus be beneficial for individualised treatment and medical decision making.

## Supplementary Information

**Additional file 1:** **Table S1.** Enrichment analysis of KEGG pathways by using GSVA.

**Additional file 2:** **Table S2.** The comparison of immune cell contents identified in the two subtypes.

**Additional file 3:** **Fig. S1.** Co-expression analysis of 1747 immune genes in the TCGA cohort. (A) Calculation of the scale-free fit index of various soft-thresholding powers and the analysis of the mean connectivity of various soft-thresholding powers. (B) Median rank and Z-summary statistics in the module preservation process. The plot shows the module position in the test dataset based on the Median rank(left). The plot illustrates the analysis of the Z-summary between different modules in the test dataset(right). (C) Heatmap of module trait relationships between gene modules and traits of THCA. The correlation coefficient in each cell represents the correlation between the modules and traits, which increases in size from blue to red. The corresponding *P*-value is annotated. The module–trait relationships were demonstrated by correlation values and *P*-values (In parenthesis) with a range of colors; the degree of correlation between modules and clinical features is shown. Rows are module eigengene (ME) regards to each module, and the columns indicate traits.

**Additional file 4:** **Table S3.** The full list of significant differentially expressed genes.

**Additional file 5:** **Table S4.** The statistical results of Unicox and Multicox analysis.

## Data Availability

The datasets generated and/or analysed during the current study are available in The Cancer Genome Atlas database (https://portal.gdc.cancer.gov/projects/TCGA-THCA, accession number phs000178) and the UCSC Xena (https://xenabrowser.net/datapages/?cohort=TCGA%20Thyroid%20Cancer%20(THCA)&removeHub=https%3A%2F%2Fxena.treehouse.gi.ucsc.edu%3A443).

## Abbreviations

PTC: Papillary thyroid carcinoma
RAI: Radioactive Iodine
THCA: Thyroid carcinoma
TCGA: The Cancer Genome Atlas
GSEA: Gene set enrichment analysis
GSVA: Gene set variation analysis
GO: Gene ontology
KEGG: Kyoto Encyclopedia of Genes and Genomes
WGCNA: Weighted correlation network analysis
ROC: Receiver operating characteristic
AUC: Area under the ROC Curve
TME: Tumour microenvironment
TNM: Tumour-node-metastasis
FC: Fold-change
