# Supplementary material for: Identification of ferroptosis genes in immune infiltration and prognosis in thyroid papillary carcinoma using network analysis
Source: BMC Genomics. 2021 Jul 27;22:576. doi: 10.1186/s12864-021-07895-6 (PMC8314640; doi:10.1186/s12864-021-07895-6)
Supplement: Supplementary file 1 — Additional file 1: Table S1. Enrichment analysis of KEGG pathways by using GSVA. [file 12864_2021_7895_MOESM1_ESM.docx]

## Supplementary figure


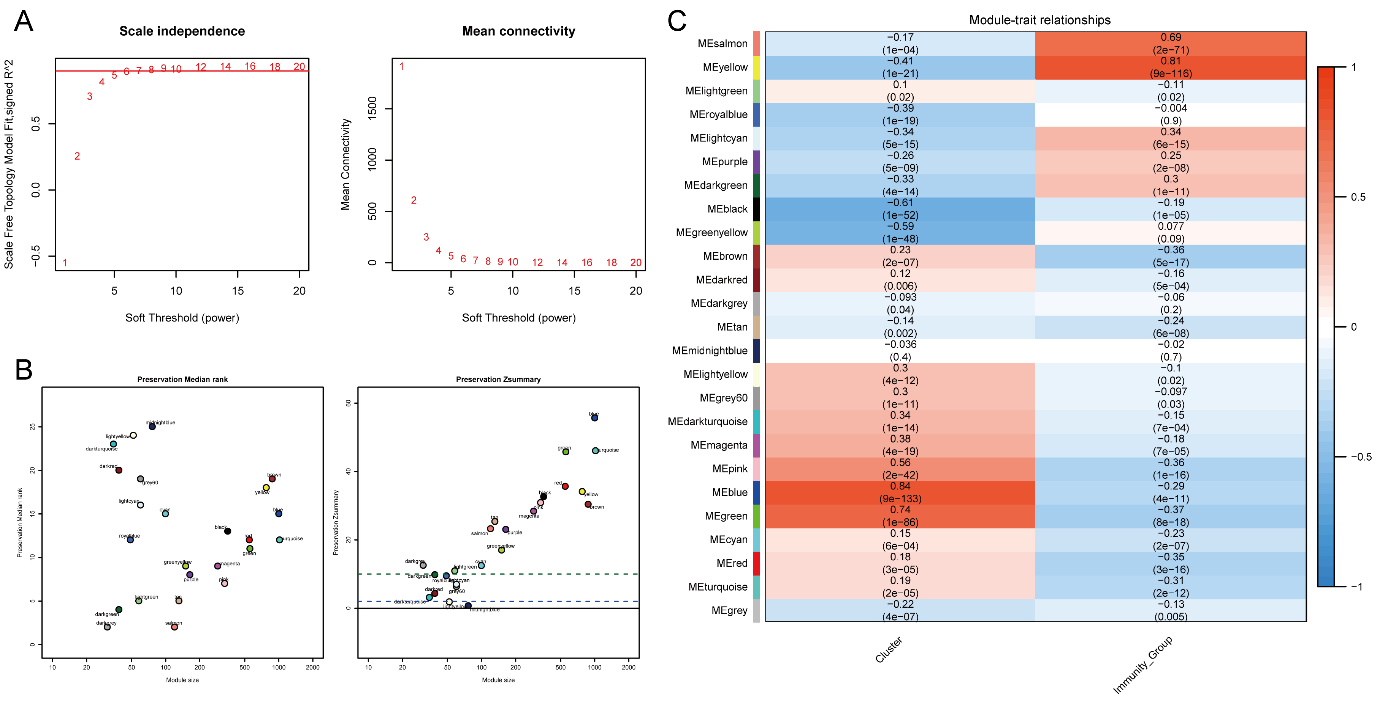


**Fig. S1** Co-expression analysis of 1747 immune genes in the TCGA cohort. (A) Calculation of the scale-free fit index of various soft-thresholding powers and the analysis of the mean connectivity of various soft-thresholding powers. (B) Median rank and Z-summary statistics in the module preservation process. The plot shows the module position in the test dataset based on the Median rank(left). The plot illustrates the analysis of the Z-summary between different modules in the test dataset(right). (C) Heatmap of module trait relationships between gene modules and traits of THCA. The correlation coefficient in each cell represents the correlation between the modules and traits, which increases in size from blue to red. The corresponding *P*-value is annotated. The module–trait relationships were demonstrated by correlation values and *P*-values (In parenthesis) with a range of colors; the degree of correlation between modules and clinical features is shown. Rows are module eigengene (ME) regards to each module, and the columns indicate traits.
